# From past to future: on-chip laser sources for photonic integrated circuits

**DOI:** 10.1038/s41377-022-01006-0

**Published:** 2023-01-15

**Authors:** Junjie Yang, Mingchu Tang, Siming Chen, Huiyun Liu

**Affiliations:** Department of Electronic and Electrical Engineering, University College London, London, WC1E 7JE United Kingdom

**Keywords:** Semiconductor lasers, Photonic devices

## Abstract

The realisation of on-chip light sources paves the way towards the full integration of Si-based photonic integrated circuits (PICs).

Driven by the substantially increased functionality of smartphones, cloud services and Internet-of-Thing devices, global internet traffic has increased explosively over the past decades. The ever-increasing demands of bandwidth significantly challenge signal processing systems, especially in data centres, where high speed, large bandwidth and low power consumption are vital concerns. Under this circumstance, the Si-based PIC, in which both optically active and passive components are monolithically integrated on a single chip, offers high bandwidth density, high energy efficiency and low latency. It is, therefore, proposed to innovate the next generation of information and communication technology^1^.

The distinct advantage of Si-based PICs is the ability to leverage mature complementary metal–oxide–semiconductor (CMOS) technology and allow mass production at a low cost. The application of Si-based PICs expands its landscape from Datacom to sensing technologies, such as automotive LiDAR, biosensors and envisioned future technologies including integrated quantum technologies, optical computing, artificial intelligence (AI)-based technologies and neuromorphic photonics. Rapid advances have been witnessed in the past decades for the key components of Si-based PICs, including high-performance Si-based modulators, photodetectors and waveguides^1^. But above all, the electrically pumped, efficient, stable light sources on Si still constitute a challenge.

An ideal on-chip Si light source shall meet the criteria based on its applications. In general, the operating conditions of the on-chip light source are: (1) Electrically pumped, continuous-wave (CW) lasing covering modern Si electronic chips operating temperatures range from -40 to 85 °C. (2) Operating with low energy consumption, sufficient-high output power and low energy cost per gigabit. (3) Operating at Telecom- and Datacom-wavelength bands such as O-band (~1310 nm) and C-band (~1550 nm) for seamless interconnection with the current fibre-optic network. (4) Directly integrating onto a Si platform compatible with mature CMOS processing technology for large-scale manufacturing.

Unfortunately, realising an efficient Si laser has been considered the “Holy Grail” for Si photonics. This is because the two widely used materials in integrated circuits, Si and Ge, are indirect bandgap materials, which make them inefficient light emitters. Although great effort has been made to realise group-IV lasers through various methods, such as Si Raman lasers, band-engineered Ge lasers and (Si)GeSn-based lasers, the high threshold current and low quantum efficiency make them fail to meet the aforementioned requirements and are far behind the practical use as on-chip light sources^2^. In contrast, direct bandgap III–V compound semiconductors have robust optical properties that can be tailored for III–V emitters operating at various wavelengths with high efficiency, large modulation bandwidth, and sufficient optical power output for many photonic applications^3^. Hence, the integration of III–V materials onto Si substrates is proposed as it leverages the benefits of both III–V (superior optical properties) and Si (large wafer size, low cost and mature processing technology).

In the recent issue of *eLight*, Prof. John Bowers, Dr. Yating Wan and colleagues delivered a comprehensive review of the state-of-the-art integrated on-chip lasers for the Si-based PICs in both device-level integration and system-level applications. They thoroughly discussed the prospects of Si-based PICs with incorporated on-chip light sources (Fig. 1)^4^. The authors first introduced the different routes for integrating III–V lasers onto Si. The primary integration methods of III–V materials on Si have been intensively explored, including hybrid integration, wafer-bonding-based heterogeneous integration and direct epitaxy. Currently, the successfully commercialised III–V lasers on Si mainly depend on the heterogeneous integration in which III–V materials are grown on the native substrates and then bonded to a pre-patterned silicon-on-insulator substrate. However, the potential issues associated with this technique remain as high cost, low yield and limited scalability.

**Fig. 1:**
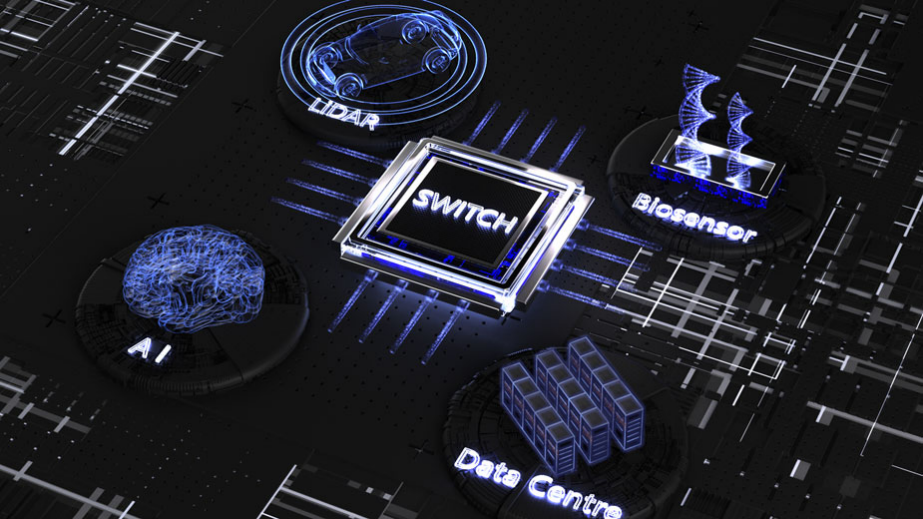
Prospects of on-chip lasers. PIC with on-chip laser sources for myriad possible applications, including LiDAR, biosensor, AI and data centre

Compared to heterogeneous integration, the direct epitaxy of III–V materials on Si substrates is attractive for integrating III–V lasers on Si substrates with low cost and high integration density. Nevertheless, the direct epitaxy of III–V materials onto Si substrates introduces a high density of crystalline defects such as threading dislocations (TDs), antiphase boundaries and micro-cracks, which form a high density of non-radiative recombination centres and significantly degrade the performance of laser devices^5^. Therefore, several solutions were applied to enhance the laser performance, especially minimising the defect density inside the laser structure to a sufficiently low value and choosing a defect-insensitive III–V gain medium, such as the quantum dot (QD) structure. Due to the delta-function-like density of states and local strain field, QDs are much less sensitive to defects when compared with quantum wells. Hence, QD-based lasers possess several advantages, including low threshold current density, temperature insensitivity and long lifetime^6,7^.

Remarkably, the authors also reviewed the merits and implementation of on-chip light sources for various ongoing applications of PICs. The breakthroughs were highlighted with a clear timeline. Currently, the most prominent driving force for further developing PICs is Datacom. Leading companies such as *Intel*, *Cisco*, and *Juniper* *Networks* offer commercialised products of PICs by implementing heterogeneous integration of on-chip laser sources^8^, e.g., *Intel* demonstrated the volume production of 100 G CWDM4 optical transceivers.

Beyond Datacom, more merging applications of PICs have also been explored with dense device integration such as integrated optical phased arrays (OPA)-based LiDAR. Various breakthroughs have been demonstrated in the Si photonics-based LiDAR system over the past decades. Especially, *Mobileye* unveiled a Si photonics-based LiDAR for autonomous vehicles at CES 2021^9^, representing a big step towards commercialisation. However, for the mass production of commercialised on-chip LiDAR system, further optimisations for the on-chip laser sources for LiDAR are required, such as cost reduction, narrow bandwidth, high-frequency modulation linearity, sufficiently-high power output and high speed of the tunning response.

A significant rise in demand for reliable, portable biosensors has happened in these years; furthermore, the Covid-19 pandemic and ageing society also stress this importance. Over the past decades, the PICs have made great progress in this area. In 2021, *Rockley Photonics* demonstrated a real-time, non-invasive, wearable biosensing platform based on Si photonics. This product supports several measurements, including heart rate, blood pressure, core body temperature, glucose indicator, etc^10^. With the further development of PICs, biosensors would realise lab-on-chip devices and promise increased functionality in the future, which significantly enhances the well-being and life quality of consumers.

The authors gave a deep insight into the applications of PICs in the envisioned technologies, including integrated quantum photonics (IQP) technology and quantum computing. To achieve a compact device size with high integration density, Si-based IQP has been intensively studied. Despite various efforts that have been taken to realise each key component, it is more realistic to realise IQP with various material systems^11^. So far, a chip-based IQP remains a scientific challenge. With a successfully integrated on-chip light source, a fully integrated Si-based IQP is promising for commercialisation in the future.

Furthermore, optical computing inspires ultra-high computation speed and ultra-low latency with ultra-low power consumption. The WDM-matrix-vector multiplication (MVM) offers a distinct advantage for ultra-dense computing in optics and is proposed for hardware platforms. Integrated comb lasers that provide thousands of coherent laser lines with low power consumption act as promising on-chip light sources for WDM-MVM photonic processors. A step further is required to co-pack on-chip lasers, micro-combs and other devices on a single chip for high-level integration, and hence enable optical computing in a better place for high-performance information processing^12^.

In summary, this paper reviewed the ongoing progress of state-of-the-art on-chip light sources and highlighted the perspects and applications of Si-based PICs with the on-chip light sources. Looking into the future, it could be expected that the direct epitaxy of Si-based III–V QD lasers provides a feasible pathway for achieving reliable, powerefficient on-chip laser sources with low cost and high integration density for Si-based PICs in the long term. While tremendous progress has been achieved in the commercialisation of Si-based PICs in optical interconnections and sensing technologies, further advances in the on-chip light sources are still demanded to enable the realisation of Si-based PICs for envisioned applications, including IQP and optical computing. The successful implementation of commercialised Si-based PICs in these fields will unleash the enormous potential and impact of these technologies on society in the foreseeable future.
